# La Neurología en Luces de Bohemia

**DOI:** 10.31083/RN37281

**Published:** 2025-01-20

**Authors:** Alfredo Puy-Núñez, Ana Guitián-Pena, Irene Expósito-Ruiz, Mercedes Macías-Arribí, Jose Manuel Aldrey-Vázquez, Juan Manuel Pías-Peleteiro

**Affiliations:** ^1^Servicio de Neurología, Complejo Hospitalario Universitario de Ferrol, 15405 Ferrol, A Coruña, España; ^2^Grupo de Estudio de Historia de la Neurología y Humanidades, Sociedade Galega de Neuroloxía, 36201 Vigo, Pontevedra, España; ^3^Servicio de Nefrología, Complejo Hospitalario Universitario de A Coruña, 15006 A Coruña, A Coruña, España; ^4^Servicio de Neurología, Complejo Hospitalario Universitario de Santiago de Compostela, 15706 Santiago de Compostela, A Coruña, España

**Keywords:** encefalitis, epilepsia, ictus, literatura, muerte, síndrome de Charles Bonnet, encephalitis, epilepsy, stroke, literature, death, Charles Bonnet syndrome

## Abstract

**Introducción:**

La obra Luces de Bohemia de Valle-Inclán es la obra inaugural del esperpento, género literario que nace con la intención de ofrecer imágenes distorsionadas de la realidad como forma de acceder a ella de manera reflexiva.

**Material y Métodos:**

Lectura crítica de la editio princeps de la obra para analizar su contenido neurológico.

**Resultados:**

El personaje de Max Estrella, inspirado en la figura de Alejandro Sawa, no cumpliría criterios de una encefalitis, siendo más probables los diagnósticos de síndrome de Charles Bonnet y fallecimiento por un ictus. Los personajes del velatorio discuten acerca del diagnóstico diferencial entre muerte y catalepsia, y uno de ellos sufre un evento no epiléptico.

**Conclusiones:**

Luces de Bohemia refleja distintos aspectos sociales, políticos y culturales contemporáneos al autor. La salud y la enfermedad también son abordados, con un papel preponderante para la neurología.

## Abstract (en)

**Introduction:**

Valle-Inclán's Bohemian Lights is the inaugural work of the esperpento, a literary genre that was born with the intention of offering distorted images of reality as a way of accessing it in a reflexive way.

**Material and Methods:**

Critical reading of the editio princeps of the work to analyze its neurological content.

**Results:**

The character of Max Estrella, inspired by the figure of Alejandro Sawa, would not meet the criteria for encephalitis. The diagnoses of Charles Bonnet syndrome and death due to stroke are more likely. The characters at the wake discuss the differential diagnosis between death and catalepsy, and one of them suffers a non-epileptic event.

**Conclusions:**

Bohemian Lights reflects different social, political and cultural aspects contemporary to the author. Health and disease are also addressed, with a preponderant role for neurology.

## 1. Introducción

Ramón María del Valle-Inclán (Vilanova de Arousa, 1866-Santiago de Compostela, 1936) es una de las figuras más destacadas de la denominada Generación del 98. Adscrito a la bohemia, participó durante años de la vida cultural madrileña durante el periodo de la Restauración, sistema con el que mantuvo una actitud muy crítica. Luces de Bohemia constituye una de las obras capitales dentro de la producción del escritor gallego en cuanto que supone la primera composición adscrita específicamente al esperpento. En las propias palabras del autor, constituye un género iniciado por el pintor Francisco de Goya y Lucientes que él inaugura en la literatura [1]. Lo describe, en un símil con los espejos cóncavos del callejón del Gato, como la toma de elementos del mundo real de los cuales nos devuelve imágenes distorsionadas, absurdas y grotescas que, tras una fase inicial de humor y extrañeza, nos muevan a una reflexión crítica del mundo. En su obra se narra el viaje por el Madrid de la Restauración de Max Estrella, escritor ciego de talento reconocido y con dificultades económicas, con su compañero de juergas y lazarillo Don Latino, su fallecimiento, velatorio y funeral, todo ello en una jornada. En su periplo se reflejan aspectos sociales, políticos, artísticos y médicos, específicamente en el campo de la neurología, como se ha apuntado en otras ocasiones [2, 3]. El objetivo de este estudio es analizar los aspectos neurológicos de esta tragicomedia.

## 2. Material y Métodos

Lectura crítica de la obra “Luces de Bohemia (esperpento)” en su editio princeps [4], con la inclusión de tres nuevas escenas (segunda, sexta y undécima) respecto a su edición original publicada por entregas semanales.

## 3. Resultados

Max Estrella, ciego por “el regalo de Venus” (según refiere en la escena octava, p. 128) se levanta animoso en su vivienda, pues ha recuperado la vista para poder ver el mundo, en este caso la Moncloa ante sí. Al perder la visión de nuevo, se reclina en el sillón entristecido y se adormece (escena primera, p. 48–50). Posteriormente, Max se ve a sí mismo y a Don Latino presidiendo el entierro de Víctor Hugo en París. Max refiere que le resulta incomprensible cómo puede volver a ver y Don Latino le replica que ya ha tenido esa misma ilusión otras veces (escena duodécima, p. 172).

Ya de madrugada (escena duodécima, p. 166–174), Max Estrella dice no poder levantarse ni tenerse en pie. Don Latino le refiere que tiene “una fisionomía algo rara” y le pide que no tuerza la boca hasta en cuatro ocasiones, a lo que Max replica que será algo nervioso porque no puede evitarlo, se queja de frío en las manos y dolor en las uñas: “mira cómo me he quedado de un aire”.

En la escena decimatercia, Max, declarado fallecido por “un colapso” resultado de no cuidarse (p. 184), es visitado en el velatorio por Basilio Soulinake, anarquista ruso que declara que su primera impresión al entrar es que no está muerto, sino que es un interesante caso de catalepsia (p. 185) como los que ha visto y estudiado en hospitales de Alemania (p. 188). Frente a la portera del edificio que comenta el olor a corrupción, esgrime como argumento de autoridad que ha estudiado medicina durante diez años, aunque no ha llegado a ser doctor, y que no acepta la opinión de la portera si carece de estudios universitarios. Ésta propone demostrar la falta de aliento aplicando un espejo a la boca (p. 187), que aquél tacha de comprobación anticientífica. Finalmente, el cochero fúnebre, apremiado por sus horarios, propone arrimar una cerilla encendida al pulgar del muerto hasta que se consuma ya que, de no haber respuesta, sin duda está muerto (p. 188). La ejecución de la maniobra provoca que la hija de Max dé un grito estridente, tuerza los ojos y comience a batir la cabeza contra el suelo mientras invoca a su padre (p. 189).

## 4. Discusión

### Aspectos Literarios de la Obra

Como creación literaria la obra tiene un claro significado simbólico: el sabio ciego que habiendo agudizado compensatoriamente otros sentidos, tiene momentos de lucidez e iluminación en relación con París (la Ciudad de la Luz en su doble significado, como capital cultural y como primera ciudad en disponer de alumbrado público) o Moncloa, juzgado como un rincón francés en Madrid. La iglesia, el frío y la noche previa al alba marcan el ambiente de tránsito entre la vida y la muerte en el último diálogo de Max Estrella. Sin embargo, dada la intención expresa del autor de mostrar en su obra una versión deformada del mundo, no es descartable una base real en el relato. A ello contribuyen abundantes referencias políticas, geográficas, sociales y la aparición de personajes como el poeta Rubén Darío o el propio álter ego de Valle-Inclán, el marqués de Bradomín.

Es notoria la identificación de Max Estrella con Alejandro Sawa Martínez (Sevilla, 1862-Madrid, 1909), una ficcionalización del escritor que ya hicieron otros literatos antes y después de Valle-Inclán, incluyendo al propio Sawa [5]. Se trataría de un escritor bohemio ciego casado con una mujer francesa y una hija adolescente, ampliamente reconocido por su talento, pero con dificultades económicas y problemas para publicar. Además de reflejarse en la obra el entorno cercano a su domicilio, hay que destacar que en la pared de la vivienda de Sawa estaba presente una lámina del entierro de Víctor Hugo [6]. La escena del funeral de Max en la que los bohemios declaran que no está muerto sino cataléptico y le queman las puntas de los dedos para valorar su respuesta al dolor está narrada por Pío Baroja con Rafael Villasús, otro trasunto de Sawa en El árbol de la ciencia; escena que Valle-Inclán versiona y amplía para reparar lo que cree una injusticia por parte de Baroja, que no simpatizaba con la vida bohemia [7]. Si bien no hay constancia de que se quemase al cadáver, sí está admitido por el propio Ernesto Bark, asistente al velatorio, que el miedo a que su amigo Sawa pudiese ser enterrado vivo le llevó a discutir con los sepultureros para esperar a que se objetivasen signos de descomposición [8]. Todos estos elementos eran conocidos por Valle-Inclán, puesto que está documentada su relación de amistad con Sawa desde 1896 y asistió tanto al velatorio como al funeral [6].

Sawa era ciego y murió en su casa envuelto en delirios a lo largo de dos semanas, achacados a un cuadro de encefalitis [9] en situación de graves dificultades económicas, si bien Valle-Inclán le concede la merced de morir lúcido en la forma de Max, como una distorsión del síndrome de Alonso Quijano en el que póstumamente Sawa puede recuperar su cordura de nuevo antes de morir definitivamente [10]. No acaban ahí las diferencias, puesto que los hechos históricos reflejados en Luces de bohemia ocurren con posterioridad a la muerte de Sawa. Así, si bien Sawa es un punto de partida, Max es un constructo literario que es vehículo de las opiniones de Valle-Inclán, tanto de su amigo fallecido hace años como de la situación política del momento, especialmente en las tres escenas añadidas para la editio princeps [7]. También resulta interesante la interpretación de Don Latino como el reflejo canalla y esperpéntico de Sawa frente al de su integridad, que encarnaría Max Estrella [5]. El encuentro de Max con Don Latino (una versión parásita, vacía y con un discurso que suele hacerse eco del primero) llevándolo en su escapada a la perdición y finalmente a la muerte, como subraya la hija del difunto en el velatorio, lo entroncaría con la tradición germana del doppelgänger [11], término acuñado por Jean-Paul Richter en 1776 que ha gozado de una gran popularidad en la literatura fantástica desde el Romanticismo. Alude al doble idéntico, en ocasiones depositario de lo malvado y oculto, cuya visión produce angustia y desasosiego por contravenir la propia identidad y las leyes naturales, y que desde el punto de vista cristiano lleva aparejada el afrontamiento de la muerte, que es cuando el sujeto humano como alma se convierte en el doble del cuerpo que abandona. Deben mencionarse las lecturas de la dupla protagonista como un reflejo esperpéntico de los Dante y Virgilo de La divina comedia o de Don Quijote y Sancho Panza [12, 13]. Por todo ello, Sawa no puede ser empleado como un modelo de verificación de las circunstancias de Max Estrella.

## 5. Aspectos Neurológicos de la Obra

### 5.1 Ceguera Adquirida y Metamorfopsias

La presencia de una ceguera por una enfermedad de transmisión sexual sugiere poderosamente una lúes [14]. El protagonista la achaca al “regalo de Venus”, una de las denominaciones para “el mal francés”, estrechamente ligado a la vida de la bohemia parisina que experimentó el personaje. Esta enfermedad, referida por primera vez en el siglo XV, iba aparejada al sexo de manera natural, convivió con gran parte de la población y era parte de la realidad cotidiana ya desde la literatura del siglo XVI, si bien su progresión en paralelo a la edad solía conllevar la decadencia física, el apartamiento de la vida pública y finalmente la pérdida de capacidades mentales y la muerte [15] al menos hasta la introducción de la penicilina. El siglo XIX contó con la afectación de figuras como Baudelaire, Nietzsche y quizás Galdós. La sífilis ocular puede ocurrir en cualquier momento de la enfermedad y es una manifestación independiente, aunque frecuentemente ligada, a la neurolúes [16]. Puede producir cualquier afectación ocular, siendo uveítis y neuritis óptica las más frecuentes. El pronóstico visual sin tratamiento es pobre, más si se asocia a coriorretinitis [17]. Otras enfermedades infecciosas por esa vía de transmisión cursan de manera más temprana o evidente, como con la Chlamydia trachomatis [18]. Max no tiene como mascota ni figura como antecedente el arañazo de un gato para pensar en una bartonellosis o toxoplasmosis [19]. No constan antecedentes familiares de ceguera. El consumo de alcohol y posible déficit nutricional suponen un riesgo para una enfermedad tóxico-carencial que se manifieste como una neuropatía óptica, o también como degeneración macular, cataratas o glaucoma [20, 21]. En este sentido, salvo las alucinaciones y la clínica sensitiva distal, no hay referencia a ninguna otra afectación que permita formular la hipótesis de una encefalopatía de Wernicke [22].

Resulta llamativo que Max emplee símiles visuales al formular la teoría del esperpento: la deformidad de la imagen en términos de forma (espejos cóncavos) o distancia (el fondo de un vaso). Más allá de recurrir a una capacidad cuya ausencia tiene muy presente, cabe la posibilidad de que el personaje haya podido sufrir metamorfopsias previamente a su pérdida definitiva de la visión, clínica que refleja un daño retiniano o coroideo con independencia de la causa [23]. Si Valle-Inclán presenció esa clínica en algún consumidor crónico de alcohol, quizás por una degeneración macular, sería algo que entraría en el terreno de lo puramente especulativo.

### 5.2 Síndrome de Charles Bonnet

Al contrario que su contrapartida real, Max Estrella se mantiene durante la jornada que retrata Luces de bohemia desplegando una intensa actividad social, económica, intelectual y, por qué no decirlo, física, dando una gran muestra de lucidez en todo momento, incluso durante su intoxicación etílica, falleciendo finalmente de forma brusca por un cuadro con focalidad neurológica aguda. En su afán de retratar un personaje trágico y dignificar a su amigo, así como de condensar la apoteosis de Max Estrella en menos de un día, resulta complicado sostener el diagnóstico de una encefalitis como en su modelo, Sawa. Dada la aparición de alucinaciones visuales complejas de manera paroxística, hecho corroborado por terceros, con cierta preservación del insight, involuntarias y aún fuera del contexto de consumo enólico, en ausencia de demencia, con un déficit visual existente y sin involucrar a otra modalidad sensorial, nos inclinamos por un síndrome de Charles Bonnet, cuyos criterios cumpliría [24, 25]. Este biólogo y filósofo suízo describió esta entidad que padecía su abuelo. El diagnóstico requiere: (1) alucinaciones unimodales visuales estereotipadas que reconocen como irreales, (2) déficit sensorial debido a cualquier lesión a lo largo de la vía óptica, (3) cognición conservada y (4) descartar otras causas de alucinaciones (como tóxicas, psiquiátricas y neurológicas).

### 5.3 Ictus

El fallecimiento brusco con una focalidad neurológica aguda es una presentación sugestiva de un ictus. Dado el consumo perjudicial de alcohol (agudo y crónico) y el previsible estrés del personaje durante la ajetreada jornada narrada, es esperable la existencia de cifras elevadas de tensión arterial; por lo tanto, la hipótesis más probable sería una hemorragia [26]. Otras hipótesis a tener en cuenta serían un ictus isquémico cardioembólico por una fibrilación auricular secundaria a consumo de alcohol [27] o una hemorragia postraumática, dada la referencia a un maltrato físico durante su detención, que el personaje denuncia como haber sido escupido y abofeteado (escena quinta, p 101; escena octava, p 129). La existencia de una lúes podría traducirse en un daño cerebral adquirido como causante de alucinaciones visuales y patología oclusiva arterial [28] aunque sería una presentación infrecuente de esta infección. El síndrome de Charles Bonnet también podría deberse a daño secundario a un ictus previo.

### 5.4 Muerte, Catalepsia y Charlatanería

A finales del XIX y principios del XX se extendió el miedo a ser enterrado vivo y despertar en la sepultura, lo que llevó a la venta de ataúdes de seguridad con mecanismos como campanas que podrían accionarse por el enterrado. Esta preocupación se vio reflejada en la literatura, dada la fascinación de los románticos por los comportamientos fuera de la norma social. Bajo la denominación de catalepsia se abordan intensos relatos fantásticos en Tennyson, Poe o Eliot que estimulaban la imaginación de sus lectores [29]. La catalepsia, definida en la época como un estado de muerte aparente, incluía un terreno no claramente delimitado entre la epilepsia, la intoxicación de sustancias y la auténtica catalepsia, que resulta un fenómeno infrecuente de manera aislada [30, 31], y es el motivo de preocupación de Ernesto Bark, por su convicción de que Sawa empleaba mórficos para alivio del dolor [8].

Es en el siglo XIX cuando el médico pasa de proceder al desahucio del paciente, dejando los últimos cuidados a cargo de la familia, a emitir un certificado legal que permita su enterramiento [32]. A partir de 1900, el Instituto Geográfico y Estadístico (posteriormente Instituto Nacional de Estadística) comienza a publicar de manera ininterrumpida el Movimiento Natural de la Población. También se adopta la clasificación de las defunciones por causa de Jacques Bertillon. Ambos hitos mejoraron la certificación y estadísticas de causa de muerte en España [33]. Por ello resulta difícil que en el Madrid de un afianzado siglo XX pudiera haber grandes dudas acerca del estado de muerte de Max Estrella. Dicho estado se basaba tradicionalmente en la demostración de ausencia de signos vitales, y se corroboraba con la aparición de signos de putrefacción. La prueba del espejo pretende demostrar la ausencia de respiración. La prueba de la llama sería inadecuada, porque en realidad evalúa la integridad de la vía aferente sensitiva y la capacidad de poder realizar una respuesta motora de retirada, no la ausencia de vida.El olor a corrupción corroboraría el estado ya diagnosticado previamente por un médico. El antecedente inmediato agudo de un daño orgánico en un órgano esencial para la vida (el cerebro) y los factores predisponentes (enolismo) nos darían el sustrato causal.

### 5.5 Episodio no Epiléptico

La hija de Max sufre una caída con desviación ocular y sacudidas cefálicas, pero conservando el nivel de consciencia y lamentándose de manera congruente con la impresión del momento, datos todos que van en contra de una crisis comicial y más a favor de un evento no epiléptico en relación con un alto impacto emocional [34].

## 6. Conclusiones

Luces de bohemia refleja de manera grotesca e impactante distintos aspectos de la sociedad de Valle-Inclán, y la salud y la enfermedad no podían estar ausentes. El caso de Max Estrella cumpliría criterios de un síndrome de Charles-Bonnet y su muerte es altamente sugestiva de un ictus. En consonancia con las inquietudes de la época, los legos en la materia discuten el diagnóstico diferencial entre catalepsia y muerte. Además, se relata un episodio no epiléptico. En suma, podemos apreciar la representación de sintomatología neurológica y disquisiciones sobre la muerte de innegable valor artístico y sociológico.

## Data Availability

No aplica, por tratarse de una revisión.

## Contribuciones de los Autores

MMA y JMPP concibieron el estudio y lo diseñaron con APN, AGP, IEP y JMAV. APN e IER adquirieron los datos. APN, AGP, JMPP y JMAV analizaron e interpretaron los datos. APN y AGP redactaron el manuscrito. Todos los autores contribuyeron a los cambios editoriales en el manuscrito. Todos los autores leyeron y aprobaron el manuscrito final. Todos los autores han participado lo suficiente en el trabajo y han acordado ser responsables de todos los aspectos del mismo.

## Aprobación Ética y Consentimiento Informado

No aplica, por no involucrar participantes humanos o animales.

## Financiación

Esta investigación no recibió financiación externa.
